# Radiosynthesis and Preliminary Biological Evaluation of ^18^F-Fluoropropionyl-Chlorotoxin as a Potential PET Tracer for Glioma Imaging

**DOI:** 10.1155/2018/8439162

**Published:** 2018-12-20

**Authors:** Jing Zhao, Yu-liang Wang, Xin-bei Li, Si-yuan Gao, Shao-yu Liu, Yu-kun Song, Jing-yan Wang, Ying Xiong, Hui Ma, Li Jiang, Zhi-yun Yang, Gang-hua Tang, Jian-ping Chu

**Affiliations:** ^1^Department of Radiology, The First Affiliated Hospital, Sun Yat-Sen University, Guangzhou 510080, China; ^2^Department of Radiology, Shenzhen City Nanshan District People's Hospital, Shenzhen 518000, China; ^3^Department of Radiology, Shenzhen Traditional Chinese Medicine Hospital, Shenzhen 518033, China; ^4^Guangdong Engineering Research Center for Medical Radiopharmaceuticals Translational Application PET-CT Center and Department of Nuclear Medicine, The First Affiliated Hospital, Sun Yat-Sen University, Guangzhou 510080, China; ^5^Department of Radiology, The First Affiliated Hospital of Xiamen University, Xiamen 361003, China; ^6^Department of Neurology, Sun Yat-Sen Memorial Hospital, Sun Yat-Sen University, Guangzhou 510120, China

## Abstract

**Purposes:**

Chlorotoxin can specifically bind to matrix metalloproteinase 2 (MMP-2), which are overexpressed in the glioma. In this work, radiosynthesis of [^18^F]-fluoropropionyl-chlorotoxin ([^18^F]-FP-chlorotoxin) as a novel PET tracer was investigated, and biodistribution in vivo and PET imaging were performed in the C6 glioma model.

**Procedures:**

[^18^F]-FP-chlorotoxin was prepared from the reaction of chlorotoxin with [^18^F]-NFB (4-nitrophenyl 2-[^18^F]-fluoropropionate), which was synthesized from multistep reactions. Biodistribution was determined in 20 normal Kunming mice. Small-animal PET imaging with [^18^F]-FP-chlorotoxin was performed on the same rats bearing orthotopic C6 glioma at different time points (60 min, 90 min, and 120 min) after injection and compared with 2-deoxy-2-[^18^F] fluoro-D-glucose ([^18^F]-FDG).

**Results:**

[^18^F]-FP-Chlorotoxin was successfully synthesized in the radiochemical yield of 41% and the radiochemical purity of more than 98%. Among all the organs, the brain had the lowest and stable uptake of [^18^F]-FP-chlorotoxin, while the kidney showed the highest uptake. Compared with [^18^F]-FDG, a low uptake of [^18^F]-FP-chlorotoxin was detected in normal brain parenchyma and a high accumulation of [^18^F]-FP-chlorotoxin was found in the gliomas tissue. The glioma to normal brain uptake ratio of [^18^F]-FP-chlorotoxin was higher than that of [^18^F]-FDG. Furthermore, the uptake of [^18^F]-FP-chlorotoxin at 90 min after injection was better than that at 60 min after injection.

**Conclusions:**

Compared with [^18^F]-FDG, [^18^F]-FP-chlorotoxin has a low and stable uptake in normal brain parenchyma. [^18^F]-FP-Chlorotoxin seems to be a potential PET tracer with a good performance in diagnosis of the glioma.

## 1. Introduction

Amongst primary brain tumors, gliomas can be considered as the most lethal malignant tumors [1, 2]. Although it is possible to roughly visualize the glioma with current imaging techniques, preoperative imaging does not always clearly define the tumor parenchyma and the edge of the tumor invasion. Positron emission tomography (PET) provides additional insights beyond magnetic resonance imaging (MRI) into the biology of gliomas. Currently, amino acid tracers have been used predominantly for glioma imaging and exhibit lower uptake in normal brain tissue, which are better suitable for delineation of tumor extent and treatment planning than ^18^F-2-fluoro-2-deoxy-D-glucose ([^18^F]-FDG) [3–7]. Among all types of amino acid tracers, S-[^11^C]methyl-L-methionine ([^11^C]-MET) and [^18^F]-FET PET are preferred for clinical use [5, 8, 9].

Some investigations have demonstrated that [^11^C]-MET had a higher sensitivity and a lower specificity varied between 75% and 100%. Unfortunately, [^11^C]-MET is not the ideal tumor tracer, since inflammatory processes are also known to show increased [^11^C]-MET uptake [5]. Moreover, unspecific [^18^F]-FET uptake has also been observed in nonspecific brain lesions [10, 11], and a lack of [^18^F]-FET uptake does not exclude a glioma, as approximately one-third of WHO grade II gliomas and most dysembryoplastic neuroepithelial tumors are [^18^F]-FET negative [12].

Due to the small size of peptides, both high target-to-background ratio and rapid blood clearance can often be achieved with radio-labeled peptides [13, 14]. Developing glioma-specific radiolabeled peptides might be helpful in glioma evaluation. Chlorotoxin is a small 36 amino acid peptide with small molecular weight and condensed molecular structure, which facilitates it cross the blood-brain barrier (BBB) [15, 16]. Recent literatures found that chlorotoxin could specifically block the chlorotoxin-sensitive chloride ion channels and/or bind to matrix metalloproteinase 2 (MMP-2) in positive tumor cells, which are overexpressed in the glioma, but they were absent or express in low abundance in healthy tissues or in tumors of nonglial origin [17–21].

Currently, investigators had successfully conjugated chlorotoxin with nanoparticles as an MRI contrast agent [22]. However, this agent failed to cross the BBB due to large molecular weight, and it gathered in the vessels gap which made the biological safety concerned [23]. Other studies [24, 25] have demonstrated that ^131^I-labeled chlorotoxin could specifically bind with glioma tumor cells and kill them in the same time. However, the low resolution of SPECT has limited its utility for glioma assessment [26]. Our team has synthesized 4-nitrophenyl-2-[^18^F]-fluoropropionate ([^18^F]-NFB), which serves as an intermediate to conjugate with chlorotoxin [27]. Furthermore, PET-CT has a better resolution and a higher sensitivity in detecting the tumor than SPECT. This novel glioma-specific PET tracer can provide a new direction for early diagnosis and boundary delineation of gliomas.

Therefore, the purpose of our study was to radiosynthesis [^18^F]-FP-chlorotoxin and to evaluate its performance in glioma imaging with the C6 glioma model.

## 2. Materials and Methods

### 2.1. General

All chemicals obtained commercially were used without further purification unless otherwise indicated. [^18^F]-FDG was prepared as previously reported [28]. Ethyl-2-bromopropionate, acetonitrile (MeCN), dimethylsulfoxide (DMSO), *N*,*N*-diisopropylethylamine (DIPEA), bis(4-nitrophenyl)carbonate (NPC), tri-fluoroacetic acid (TFA), and Kryptofix 222 (K2.2.2) were purchased from Sigma-Aldrich (Milwaukee, WI, USA). Sep-Pak light QMA cartridges, Sep-Pak plus C18 cartridges, and Oasis HLB cartridges were achieved from Waters Corporation (Milford, MA, USA). Sep-Pak light QMA cartridges were preconditioned with 5 ml NaHCO_3_ aqueous (8.4%) and 10 ml H_2_O before using. Sep-Pak plus C18 and Oasis HLB cartridges were preconditioned with 10 ml ethanol and water, respectively, before using. High-performance liquid chromatography (HPLC) separation was performed on at the PET-MF-2V-IT-1 synthesizer (PET Co. Ltd., Beijing, China) building HPLC system with a semipreparative reverse-phase C18 column (106 and 250 mm) equipped with a UV detector (Alltech 201, USA) and a radioactivity detector (PET Co. Ltd., China). The mobile phase was 50% solvent A (0.1% TFA in water): 50% solvent B (0.1% TFA in MeCN) with the flow rate of 4 ml/min.

### 2.2. Cell Culture and Animal Models

The C6 glioma cell line, Kunming mice, and specific pathogen-free SD rats 6–8-week-old and 200–250 grams in weight were obtained from the Laboratory Animal Centre of Sun Yat-Sen University. The cells were cultured in the 1640 medium with a physiologic glucose concentration (1.0 g/l) containing 10% fetal calf serum at 37°C in a humidified atmosphere of 5% CO_2_ and 95% air.

SD rats were anesthetized by 2% pentobarbital solution (0.225 ml/100 g). The skin of the head was sterilized with iodine, and the skull was exposed after incision. A hollow guide screw was implanted into a small drill hole made 3 mm right lateral and 1 mm anterior to the bregma point. C6 glioma cells (1 × 10^6^) grown in RPMI-1640 medium supplemented with 10% fetal bovine serum were injected through the hollow guide screw into the white matter slowly in 5 min at a depth of 5 mm. After 5 min, the drilled hole was sealed with bone wax to prevent any reflux. The wound was sutured and covered with surgical glue. The whole procedure was performed under sterile conditions. Experiments were approved by the Institutional Animal Care and Utilization Committee (IACUU) of the First Affiliated Hospital of the Sun Yat-sen University (approval no. IACUC-DB-15-1002). All efforts were made to minimize animal suffering, to reduce the number of animals used, and to utilize alternatives to in vivo techniques, if available.

### 2.3. Semiautomated Radiosynthesis of [^18^F]-FP-chlorotoxin

The automated synthesis of [^18^F]-NFP was carried out on the PET-MF-2V-IT-1 synthesizer through three-step, one-pot procedure as described in detail in our previous study [29]. The starting activity of the F-18-fluoride is about 1110–1850 MBq (30–50 mCi). Vial B1 was filled with 1 ml K2.2.2 (15 mg of Kryptofix 2.2.2, 3 mg of K_2_CO_3_, 0.9 ml of acetonitrile, and 0.1 ml of water). Vial B2 was filled with 1.5 ml anhydrous MeCN, Vial B3 was filled with 1 ml ethyl 2-bromopropionate (5 mg in 1 ml MeCN), Vial B4 was filled 0.22 ml 0.2 M potassium hydroxide (KOH) solution and 0.5 ml MeCN, Vial B5 was filled with 1.2 ml NPC (40 mg in 1.2 ml anhydrous MeCN), Vial B6 was filled with 5% acetic acid in water (1 ml), Vial B10 was filled 0.1% TFA in water (40 ml), and Vial B12 was filled with ether (5 ml). Vial B7 was filled with 0.5 mg chlorotoxin, 200 *μ*L DMSO, and 20 *μ*L DIPEA. Vial B8 was filled with 5% acetic acid in water (10 ml). Vial B9 was filled with ethanol.

After the delivery of ^18^F-fluoride from the cyclotron, the radioactivity was through a Sep-Pak light QMA cartridge, where [^18^F]-fluoride was trapped. The trapped fluoride ion (^18^F-) was eluted off the Sep-Pak QMA cartridge into the vessel with a solution of K2.2.2 (Vial B1). The reaction mixture was evaporated under a stream of nitrogen (80 ml/min) at 100°C, and it took about 10–11 mins. Then, the residue was azeotropically dried again at 116°C with MeCN (Vial B3 1.5 ml MeCN). Ethyl 2-bromopropionate in anhydrous MeCN (Vial B3) was added to the vessel 1. The reaction mixture was heated at 100°C for 10 min. The ethyl 2-^18^F-fluoropropionate was hydrolyzed to the 2-^18^F-fluoropropionate salt by using the 0.2 M KOH solution (Vial B4) and heating the mixture at 100°C for 10 min. The reaction mixture was evaporated under a stream of nitrogen. Then, NPC solution (Vial B5) was added to the vessel 1 and heated at 100°C for 10 min. After cooling, 5% acetic acid (Vial B6) was added to the vessel 1 for washing out the reaction and transferring to B0. Then, the reaction mixture was purified by the in-built semi-HPLC. The ^18^F-NFP was collected into Vial B10, which was containing 40 ml water with 0.1% TFA, and then was adsorbed in the Sep-Pak Plus C18 cartridge. After cartridge was dried by nitrogen, the [^18^F]-NFP (**9**) was eluted off the cartridge by the either and further passed through two anhydrous sodium sulfate (Na_2_SO_4_) cartridges into vessel 2. The solution was removed with a stream of nitrogen at room temperature.

Anhydrous 9 was added into a mixture solution of chlorotoxin (0.5 mg, Peptide Institute, Inc. Japan) (Vial B7). The reaction mixture was heated for 10 min at 40°C, was quenched by adding 5% acetic acid (Vial B8), and was diluted with water (10 ml). It was passed through two Oasis plus HLB cartridges followed by washing with water (2 ml). [^18^F]-FP-Chlorotoxin trapped on two Oasis plus HLB cartridges was eluted with ethanol (2 ml) into a new vessel instead of vessel 2. The solvent was then removed by a stream of nitrogen at 70°C. The final product [^18^F]-FP-chlorotoxin was formulated in 0.9% saline and passed through a 0.22 mm Millipore filter for next studies.

### 2.4. Determination of Radiochemical Purity

Similar to our previous study [30], the identity of [^18^F]-FP-chlorotoxin was confirmed by analytical HPLC to determine its chemical purity. The standard [^18^F]-FP-chlorotoxin was injected into the HPLC to verify the component of [^18^F]-FP-chlorotoxin. Analytical HPLC was performed using an Agilent 1200 Series HPLC system equipped with a ZORBAX Eclipse XDB-C18 analytic column (4.66150 mm and 5 mm) using the flow rate of 1 ml/min. The gradient program started from 98% solvent A (0.1% TFA in water): 2% solvent B (0.1% TFA in MeCN) ramped to 90% solvent A: 10% solvent B at 8 min and ramped to 20% solvent A: 80% solvent B at 20 min. The elution profile was detected with an ultraviolet detector (Agilent interface 35900E, Agilent Technologies, USA) at 210 nm and a B-FC-3200 high energy PMT Detector (Bioscan. Inc., Washington DC, USA).

### 2.5. Biodistribution of [^18^F]-FP-chlorotoxin

The mice were anesthetized with 2% pentobarbital solution (0.225 ml/100 g) before injection of radiotracer and remained anesthetized through the study. Similar to our previous study [30], normal Kunming mice were injected with (0.7–0.8) MBq (20–23 *μ*Ci) of [^18^F]-FP-chlorotoxin in 0.2 ml of saline through the tail vein. Radioactivity in the syringe before and after administration was measured in a calibrated ion chamber. Mice were sacrificed by cervical dislocation at various times (10 min, 30 min, 60 min, and 120 min) after injection. Blood was obtained through the eyeball vein, and the organs of interest (the brain, heart, lung, liver, kidney, pancreas, spleen, stomach, intestine, muscle, and bone) were rapidly dissected and weighed. ^18^F-radioactivity was counted with a *γ*-counter (SN-6105, Shanghai Nuclear Rihuan Photoelectric Instrument LLC, China). All measurements were background-subtracted and decay-corrected to the time of injection and then averaged together.

### 2.6. Small-Animal PET-CT Imaging

Following our previous study [30], the tumor-bearing rats and one tumor-free rat were kept fasting for 8 h and were scanned by the small-animal PET/CT scanner (Alibra, Bruker), in which (6.7–9.3) MBq (180–250 *μ*Ci) of [^18^F]-FP-chlorotoxin was injected via the tail vein. Ten minutes before scanning, rats were anesthetized with 2% pentobarbital solution (0.225 ml/100 g). Rats were visually monitored for breathing and any other signs of distress throughout the entire imaging period. Fifteen-minute static PET images were acquired at three-time points (60, 90, and 120 min) of after injection. For a comparative study, the same rats were scanned with [^18^F]-FDG (7.4–9.3) MBq (200–250 *μ*Ci) at 60 min after intravenous injection in the next day.

The PET-CT images were reconstructed, and regions of interest (ROIs) were drawn over the tumor and normal brain parenchyma on decay-corrected coronal images using the Alibra PET System and PMOD version 3.7 software (PMDO technologies, Zurich, Switzerland). The radioactivity concentration (accumulation) within a tumor or normal brain parenchyma was obtained from mean pixel values within the multiple ROIs volume, which were converted to kBq/cc by using a conversion factor. Assuming a tissue density of 1 kBq/cc, ROIs were converted to kBq/cc and then divided by the administered activity to obtain an imaging ROI-derived % ID/cc. We converted CT numbers (Hu) into the density of the liver or the mass and then counted the average of ROI-derived % ID/cc with them equal the value of % ID/g.

### 2.7. Western Blot Analysis

Cell lysates (25 *μ*g) from the activity assay were used for testing PAH protein expression by western blot. The blot was blocked with 5% nonfat milk in Tris-Buffered Saline and Tween 20 (TBST) and then incubated with primary antibody solution: anti-PAH (Merck Millipore, MA, USA), anti-Flag (Sigma-Aldrich, MO, USA), anti-Myc-tag (Cell Signaling, MA, USA), and anti-*β*-actin (Santa Cruz Biotechnology, CA, USA) overnight at 4°C. After washing with TBST, the membrane was incubated with goat anti-mouse IgG-HRP (Santa Cruz Biotechnology, CA, USA) for 1 h at room temperature. Signals were detected with the Super Signal West Pico Chemiluminescent Substrate (Thermo Scientific, MA, USA). This part was followed the methods of Shen et al. [31].

### 2.8. Immunofluorescent Staining

After cryosection, 2 *μ*m thick brain sections were mounted onto glass slides. Sections were washed with PBS (pH 7.4), followed by antigen retrieval using sodium citrate buffer, pH 6, at 95°C for 25 min in a water bath. Nonspecific staining in sections was blocked using 10% donkey serum in 0.1% Tween 20-PBS for 1 h at room temperature. Primary antibodies were added overnight at a dilution of 1 : 350 for goat Iba-1 (Abcam), 1 : 1,000 for rabbit MMP-2 (Abcam) at 4°C. Alexa 488-conjugated donkey anti-goat IgG (1 : 200, Jackson Lab, Suffolk, United Kingdom) or Cy3-conjugated donkey anti-rabbit IgG (1 : 200, Jackson Lab) was subsequently applied. The nuclei were counterstained with DAPI (Sigma-Aldrich). Images were taken using a microscope (Axio Imager Z1, Carl Zeiss, China). This part was similar with the methods of Hu et al. [32].

## 3. Results and Discussion

### 3.1. Radiosynthesis Results

**Figure 1:**
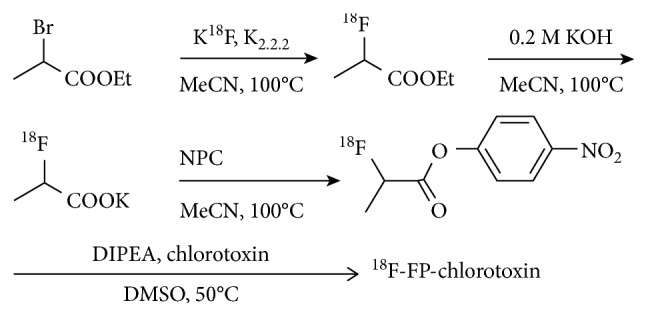
Radiosynthesis scheme of [^18^F]-FP-chlorotoxin.

**Figure 2:**
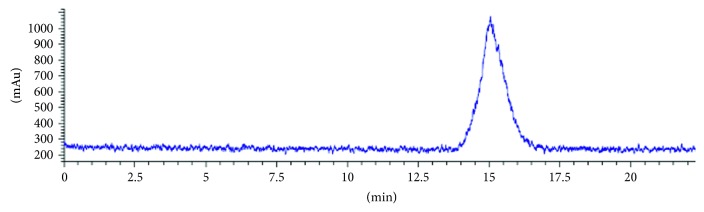
HPLC chromatograms of [^18^F]-FP-chlorotoxin.

The radiosynthesis scheme of [^18^F]-FP-chlorotoxin is shown in Figure 1. The automatic synthesis of [^18^F]-NFP was completed in 80 min with an overall radiochemical yield of 23 ± 5% (*n*=5) with decay correction. Furthermore, the manual synthesis of [^18^F]-FP-chlorotoxin was completed in 20 min with an overall radiochemical yield of 41% (*n*=5) with decay correction. The specific activity of [^18^F]-FP-chlorotoxin is greater than 3.2 × 10^−1^ GBq/*μ*mol, and the radiochemical purity of [^18^F]-FP-chlorotoxin was more than 98% (Figure 2). Radiochemical purity of [^18^F]-FP-chlorotoxin in PBS and FBS at 37°C for two hours was above 95%, showing that [^18^F]-FP-chlorotoxin in PBS and FBS kept good stability.

### 3.2. Biodistribution in Mice

**Figure 3:**
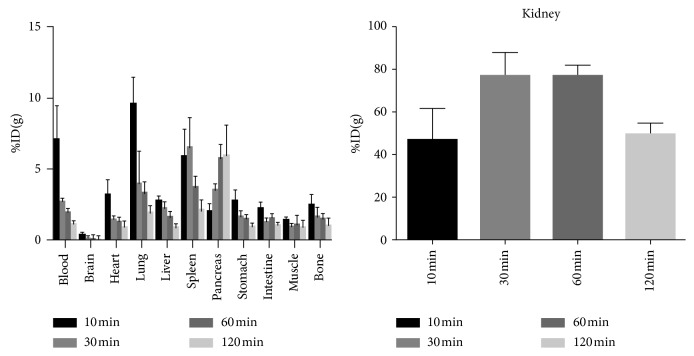
Biodistribution of [^18^F]-FP-chlorotoxin in normal mice.

The biodistribution description of [^18^F]-FP-chlorotoxin in normal Kunming mice is summarized in Figure 3. The radioactivity in the most tissues (such as blood, heart, and pulmonary) cleared fast. However, the pancreas demonstrated a moderate and increasing uptake of [^18^F]-FP-chlorotoxin during the observed time. Furthermore, the normal brain parenchyma had the lowest uptake of [^18^F]-FP-chlorotoxin over the whole observed time, and the maximal uptake value ((0.37 ± 0.10) % ID/g) was found at 10 min after injection. The highest uptake of [^18^F]-FP-chlorotoxin in all tissues at the different time points was the kidney, which was significantly higher than that in other organs. In addition, the uptake in the kidney rose sharply in the first 30 min and came to plateau at 30–60 min after injection, and then, it gradually declined.

### 3.3. PET Studies

**Figure 4:**
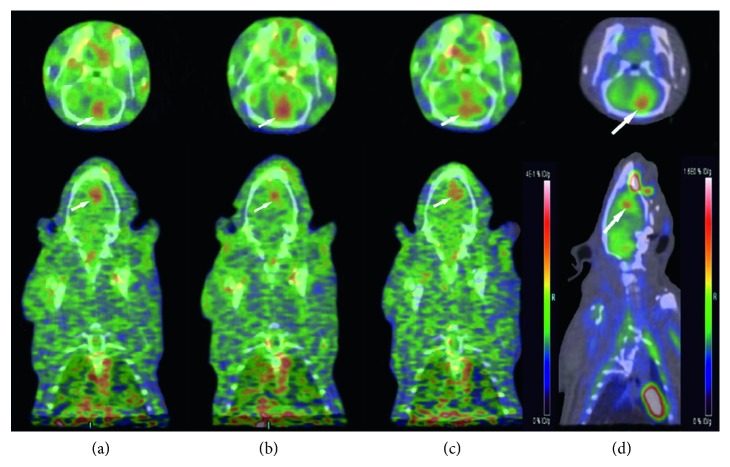
(a–c) A tumor-bearing rat with [^18^F]-FP-chlorotoxin PET examination showing that the tumor had a high accumulation of this new PET tracer with the highest uptake at 90 min after injection. (d) [^18^F]-FDG PET examination of the same rat demonstrating hypermetabolic foci in the tumor area. (a) 60 min. (b) 90 min. (c) 120 min. (d) 60 min.

Two tumor-bearing rats were scanned by small-animal PET-CT under [^18^F]-FP-chlorotoxin and [^18^F]-FDG sequentially. Compared with the normal brain parenchyma, the uptake of [^18^F]-FP-chlorotoxin in the tumor tissue was obviously higher. The average values of the tumor to brain parenchyma ratio were 1.93, 2.93, and 3.11 (Figure 4) at 60 min, 90 min, and 120 min after injection of [^18^F]-FP-chlorotoxin, respectively. However, the uptake ratio of [^18^F]-FDG in the tumor to brain parenchyma (1.44) was lower than that of [^18^F]-FP-chlorotoxin at any observed time points.

**Figure 5:**
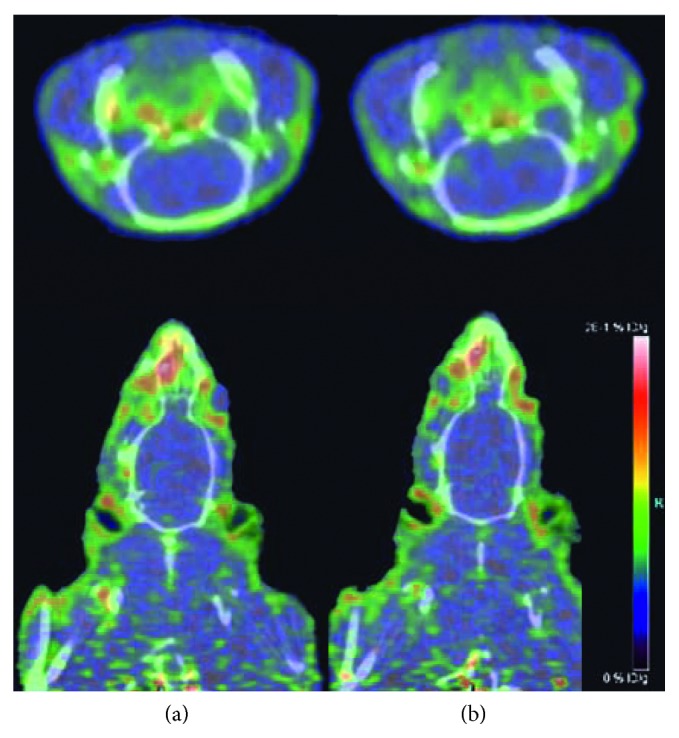
(a, b) A tumor-free rat under [^18^F]-FP-chlorotoxin PET examination showing the normal brain with consistent and lower [^18^F]-FP-chlorotoxin uptake. (a) 60 min. (b) 90 min.

Tumor-free rat under [^18^F]-FP-chlorotoxin PET-CT examination, at 60 min and 90 min, showed that the normal brain had consistent and lower [^18^F]-FP-chlorotoxin uptake (Figure 5).

### 3.4. Western Blot and Immunofluorescent Staining Results

**Figure 6:**
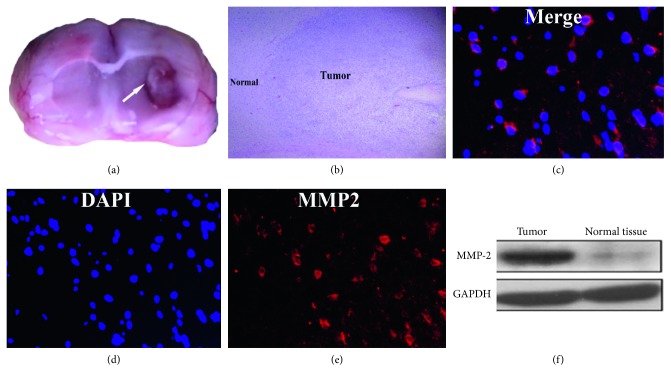
(a) The gross pathology of the glioma in the rat brain; (b) HE staining showing a clear boundary between tumor parenchyma and normal brain tissue; (c–e) the immunofluorescence image showing the MMP-2 expression in C6 glioma cells; (f) the western blot analysis showing that MMP-2 was upregulated in the tumor, compared with normal brain parenchyma.

Western blot analysis showed that the band density areas of MMP2 in the tumor area were more expressed than those in normal brain parenchyma. Slices from the glioma were immunohistologically labeled. Glioma tumor cells were identified by the expression of MMP-2 by immunolabeling in red (Figure 6).

## 4. Discussion

In this study, we successfully synthesized a novel PET tracer ([^18^F]-FP-chlorotoxin). [^18^F]-FP-chlorotoxin was prepared by two-step radiosynthesis from the reaction of [^18^F]-NFP with chlorotoxin, with a moderate radiochemical yield of 41%, and a high radiochemical purity of 98%. Semiautomated radiosynthesis had advantages (such as low radioactivity exposure and good reproducibility) over the manual operation. Moreover, the synthesis of [^18^F]-FP-chlorotoxin through conjugation of [^18^F]-NFP and lysine amino of chlorotoxin probably does not change the condensed spatial structure of the chlorotoxin, which facilitates it to cross the tumor-brain barrier. Although the ^18^F-radiolabeling process was semiautomated radiosynthesis and the radiochemical yield of [^18^F]-FP-chlorotoxin was quite stable, the radiosynthesis time was relatively long (100 min). The modification of chlorotoxin with DOTA (1,4,7,10-tetraazacyclododecane-1,4,7,10-tetraacetic acid) for ^68^Ga-radiolabeling will therefore be a high priority for our future studies, which aimed at the effort to simplify the synthesis process and reduce synthesis time.

Biodistribution experiment in vivo showed that [^18^F]-FP-chlorotoxin was quickly cleared in the blood (from 10 min: 7.05 ± 2.45% ID/g to 120 min: 1.26 ± 0.15% ID/g). The highest uptake and the following gradually decline in the kidney suggesting that the urinary system was the main excretory way. The normal brain has the extremely lowest accumulation compared to other organs. Unexpectedly, we found that the pancreas had a gradual increased uptake of [^18^F]-FP-chlorotoxin which might be due to the specific binding of [^18^F]-FP-chlorotoxin to MMP-2 expressed by the pancreatic cell. The literature indicated that 10% of pancreatic cells could express MMP-2 [21].

Our study manifested that, compared with [^18^F]-FDG, the C6 glioma rat under [^18^F]-FP-chlorotoxin examination had a better tumor-background contrast. However, tumor-free rat had no obvious uptake of [_18_F]-FP-chlorotoxin. The uptake of [^18^F]-FP-chlorotoxin by brain tumors may involve the enhanced permeability and retention (EPR) effects [33]. Based on the dynamic contrast-enhanced (DCE) MRI of C6 glioma, we found that a higher *K* ^trans^ (capillary permeability measuring, which thought it was representative of BBB) value in the tumor than in contralateral normal brain parenchyma, especially for the enhancement part in T_1_WI-enhanced MRI, which indicated that the C6 glioma tumor had provoke the BBB (Supplementary Figure 1). Furthermore, we probably assumed that [^18^F]-FP-chlorotoxin went through the destroyed brain-blood barrier and interacted with the glioma-specific ligands such as ClC-3 chloride channel, MMP-2, and *α*_V_ *ß*_3_ that caused the aggregation of chlorotoxin in the glioma [34]. Our western blot analysis did show that MMP-2 was upregulated in the tumor area and not in normal brain parenchyma. Moreover, the immunofluorescence images further confirmed the MMP-2 expression in C6 glioma cells.

The uptake of [^18^F]-FP-chlorotoxin at 90 min was higher than that at 60 min, and the uptake volume of [^18^F]-FP-chlorotoxin was increased as well. We proposed that the optimal imaging time for [^18^F]-FP-chlorotoxin was 90–120 min. Compared with [^18^F]-FDG, [^18^F]-FP-chlorotoxin had a better tumor to brain uptake ratios in the C6 orthotopic glioma rats.

Without performing the competitive inhibition, the experiment of [^18^F]-FP-chlorotoxin in vivo was the main limitation of our study. However, (1) [^18^F]-NFP has a good metabolic stability in vivo and has a few influence on the nature of the polypeptide itself [35, 36]. [^18^F]-NFP have been used in the radiolabeling of numerous peptides for PET imaging [28, 37–39]. (2) compared with [^18^F]-FDG, [^18^F]-FP-chlorotoxin had a better tumor to brain uptake ratios in the C6 orthotopic glioma rats. The interaction of chlorotoxin with glioma-specific ligands caused the aggregation of chlorotoxin in the glioma. In the tumor area, our data proved that glioma destroy the BBB, in contrast, the normal brain without obvious uptake of [^18^F]-FP-chlorotoxin. Furthermore, the immunofluorescence image showed that the MMP-2 expression in C6 glioma cells and the western blot analysis showed that MMP-2 was upregulated in the tumor compared with normal brain parenchyma. Thus, we might probably defer that [^18^F]-NFP does not modify the spacial structure of the chlorotoxin. The other limitation was the lack of the metabolization study in vitro and vivo. However, we have measured 12 ROIs in the bone and muscle of each rat at various time points, and the data demonstrated that [^18^F]-FP-chlorotoxin was stable (Supplementary Figure 2).

## 5. Conclusions

We have successfully designed and synthesized a novel polypeptide PET tracer [^18^F]-FP-chlorotoxin with a moderate radiochemical yield (41%) and radiochemical purity (>98%). Compared with [^18^F]-FDG, [^18^F]-FP-chlorotoxin shows a higher tumor uptake and a higher tumor-to-brain ratio in the glioma-bearing rat, which provides new insights into glioma imaging and tumor boundary delineation.

## Data Availability

The data of this study are already presented in the paper.

## Supplementary Materials

Supplementary Materials Supplementary Figure 1: MRI T2WI (A), susceptibility weighted imaging (SWI) (B), and conventional T1WI contrast enhancement (C) showing a soft tissue mass in the left basal ganglia. The tumor on T2WI manifested as uneven high signal, and, inside and around tumor. SWI demonstrated points and strip-shaped low signal shadows in the tumor (arrows); furthermore, the tumor showed obvious uneven enhancement. *K* ^trans^ (D, E) maps demonstrating a higher *K* ^trans^ value in the tumor than in the contralateral normal brain parenchyma, especially for the enhancement part in T1WI enhanced MRI (yellow circle: ROI 3); concentration-time curve (F) clearly shows that *K* ^trans^ in the tumor (ROI 1–3) was significantly higher than that of the contralateral normal brain parenchyma (ROI 4). Supplementary Figure 2: (a), the uptake of [^18^F]-FP-chlorotoxin in the bone with little standard deviation among the three time points, and its range is from 0.0978 to 0.1722% ID/g, which in the other way showed that [^18^F]-FP-chlorotoxin was stable; (b), the uptake of [^18^F]-FP-chlorotoxin in the muscle of each rat with little variance at 90 min.
