# Supplementary material for: Radiosynthesis and Preliminary Biological Evaluation of 18F-Fluoropropionyl-Chlorotoxin as a Potential PET Tracer for Glioma Imaging
Source: Contrast Media Mol Imaging. 2018 Dec 20;2018:8439162. doi: 10.1155/2018/8439162 (PMC6317094; doi:10.1155/2018/8439162)
Supplement: Supplementary Materials — Supplementary Figure 1: MRI T2WI (A), susceptibility weighted imaging (SWI) (B), and conventional T1WI contrast enhancement (C) showing a soft tissue mass in the left basal ganglia. The tumor on T2WI manifested as uneven high signal, and, inside and around tumor. SWI demonstrated points and strip-shaped low signal shadows in the tumor (arrows); furthermore, the tumor showed obvious uneven enhancement. K trans (D, E) maps demonstrating a higher K trans value in the tumor than in the contralateral normal brain parenchyma, especially for the enhancement part in T1WI enhanced MRI (yellow circle: ROI 3); concentration-time curve (F) clearly shows that K trans in the tumor (ROI 1–3) was significantly higher than that of the contralateral normal brain parenchyma (ROI 4). Supplementary Figure 2: (a), the uptake of [18F]-FP-chlorotoxin in the bone with little standard deviation among the three time points, and its range is from 0.0978 to 0.1722% ID/g, which in the other way showed that [18F]-FP-chlorotoxin was stable; (b), the uptake of [18F]-FP-chlorotoxin in the muscle of each rat with little variance at 90 min. [file 8439162.f1.pdf]

Supplementary Materials

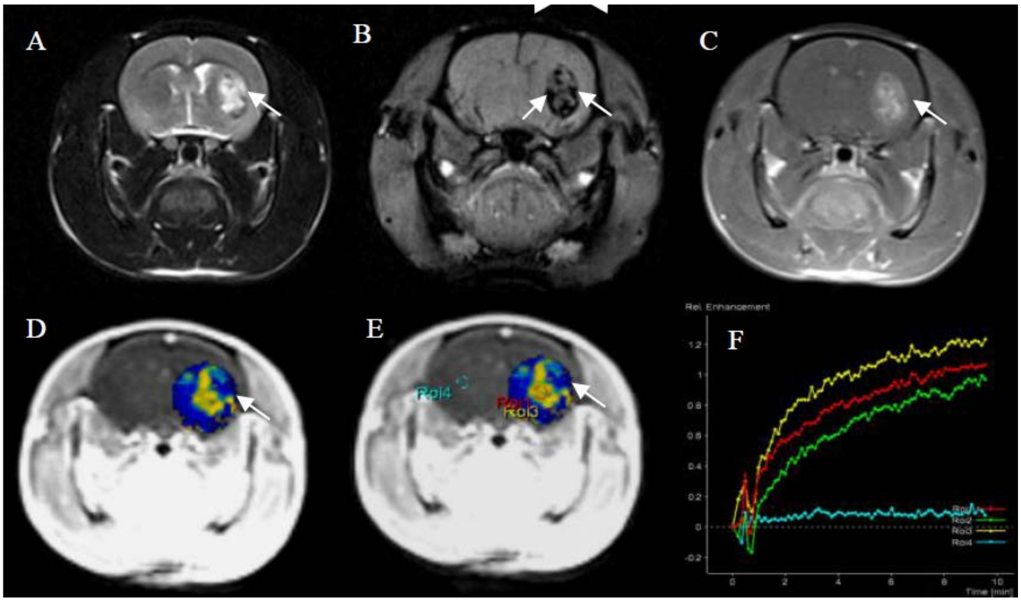

Supplementary Figure 1: MRI T2WI (A), susceptibility weighted imaging (SWI) (B), and conventional T1WI contrast enhancement (C) showing a soft tissue mass in the left basal ganglia. The tumor on T2WI manifested as uneven high signal, and, inside and around tumor. SWI demonstrated points and strip-shaped low signal shadows in the tumor (arrows); furthermore, the tumor showed obvious uneven enhancement. K^{trans} (D, E) maps demonstrating a higher K^{trans} value in the tumor than in the contralateral normal brain parenchyma, especially for the enhancement part in T1WI enhanced MRI (yellow circle: ROI 3); concentration-time curve (F) clearly shows that K^{trans} in the tumor (ROI 1-3) was significantly higher than that of the contralateral normal brain parenchyma (ROI 4).

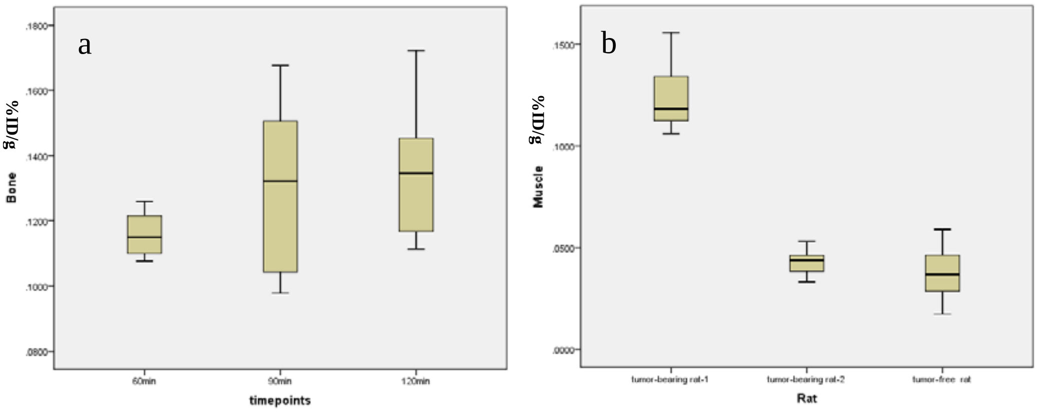

Supplement Figure 2: (a), the uptake of [^{18}F]-FP-Chlorotoxin in bone with little standard deviation among the three time points, and its range is from 0.0978 to 0.1722 %ID/g, which in the other way showed that [^{18}F]-FP-Chlorotoxin was stable; (b), the uptake of [^{18}F]-FP-Chlorotoxin in the muscle of each rat with little variance at 90 min.
